# *Streptomyces sanguinis* sp. nov., a Ceftriaxone‐Resistant Strain Obtained From Human Blood and Reclassification of *Streptomyces thermospinosisporus* as *Streptomyces thermocarboxydovorans* subsp. *thermospinosisporus* subsp. nov

**DOI:** 10.1155/ijm/5214822

**Published:** 2026-10-02

**Authors:** Imen Nouioui, Jacqueline Wolf, Meina Neumann-Schaal, Christopher A. Gulvik, John R. McQuiston, Brent A. Lasker

**Affiliations:** ^1^ Leibniz Institute DSMZ German Collection of Microorganisms and Cell Cultures Braunschweig Germany dsmz.de; ^2^ Bacterial Special Pathogens Branch Division of High-Consequence Pathogens and Pathology National Center for Emerging and Zoonotic Infectious Disease Centers for Disease Control and Prevention Atlanta Georgia USA cdc.go.kr

**Keywords:** biosynthetic gene clusters, pathogens, systematics, virulence factors

## Abstract

The taxonomic position of an actinomycete, strain 15‐116A^T^, isolated from a blood culture of a 31‐year‐old male from the United States was determined using polyphasic taxonomic analysis. Genome‐based phylogeny showed that the strain was closely related to *Streptomyces composti*, *Streptomyces thermocarboxydovorans*, and *Streptomyces thermospinosisporus* species. Digital DNA–DNA (dDDH) relatedness and average nucleotide identity (ANI) between the draft genome sequence of the strain and those of its closest phylogenomic neighbors listed above were between 35.9% and 37.3% (< 70%) and 88.5%, 88.1%, and 88.6% (95%–96.7%), respectively. However, the dDDH and ANI values between the genome sequence of *S. thermocarboxydovorans* JCM 10367^T^ and *S. thermospinosisporus* JCM 11756^T^ were 72.3% and 96.7%, respectively. These values are above the defined thresholds for prokaryotic species demarcation (70% dDDH and 95%–96.7% ANI) and fall within the dDDH range of 70%–79% for subspecies designation. Based on these findings and in accordance with the priority rule in nomenclature, *S. thermospinosisporus* is considered a later heterotypic synonym of *S. thermocarboxydovorans* and is classified as a subspecies of *S. thermocarboxydovorans*, for which the name *S. thermocarboxydovorans* subsp. *thermospinosisporus* subsp. nov. is proposed. Consequently, *S. thermocarboxydovorans* subsp. *thermocarboxydovorans* is created. Strain 15‐116A^T^ was characterized by the presence of *LL*‐diaminopimelic acid in its peptidoglycan; diphosphatidylglycerol, phosphatidylethanolamine, and phosphatidylinositol as the major phospholipids; MK‐9(H_8_) and MK‐9(H_6_) as the predominant menaquinones (> 10%); and *iso-*C_15:0_, *anteiso*‐C_15:0_, *iso-*C_16:0_, and *anteiso-*C_17:0_ as the major fatty acids (> 10%). Morphological, phenotypic, and genomic traits distinguished strain 15‐116A^T^ from its closest phylogenetic neighbors and highlighted the assignment of strain 15‐116A^T^ (= DSM 106436^T^ = ATCC BAA‐2935^T^) to a novel species of the genus *Streptomyces*, for which the name *Streptomyces sanguinis* sp. nov. is proposed. Genome screening for virulence factor‐associated genes showed that the strain harbored a gene encoding isocitrate lyase, which is required for persistent infection of *Mycobacterium tuberculosis* (YP_177728), as well as gene clusters associated with phenazine biosynthesis (NP_252902), unlike its close relatives. Moreover, strain 15‐116A^T^ possessed a gene encoding elongation factor Tu (WP_003028672), which plays an important role in the bacterium′s ability to adhere to host cells. However, further experimental studies are required to evaluate the pathogenic potential of this strain.

## 1. Introduction

Waksman and Henrici [1] proposed the genus *Streptomyces* in 1943 and are classified within the family Streptomycetaceae and order Kitasatosporales [2, 3]. Presently, the genus *Streptomyces* composes the largest known bacterial genus with over > 800 validly named species according to List of Prokaryotic names with Standing in Nomenclature (LPSN [https://lpsn.dsmz.de/], accessed June 2026) [4]. Members of the bacterial genus *Streptomyces* are characterized as aerobic, Gram‐positive, and nonmotile, with the ability to form branched substrate vegetative hyphae and spore forming aerial hyphae with the presence of *LL*‐diaminopimelic acid and glycine devoid of a characteristic sugar in their cell walls [5, 6]. *Streptomyces* are of interest due to being an important source of antimicrobials and other bioactive compounds/metabolites. Most strains have been acquired from soil, decayed vegetation, or organic matter [2, 6]. However, *Streptomyces* are a rare source of human infections with the majority of clinical manifestations presenting as suppurative granulomatous inflammation of the skin primarily by *Streptomyces somaliensis* and *Streptomyces sudanensis* [7, 8].

Herein, the present investigation reports on strain 15‐116A^T^, recovered from a clinical diagnostic blood sample obtained from a male patient in a hospital in Georgia (GA), United States. The strain was submitted to the GA Department of Public Health Laboratory by the hospital, and the strain then submitted to the Special Bacteriology Reference Laboratory (SBRL) at the Centers for Disease Control and Prevention (CDC), Atlanta, Georgia, United States for identification. The strain was shown to be a novel species belonging to the genus *Streptomyces* based on a polyphasic approach encompassing morphological, phenotypic, chemotaxonomic, and genomic analyses.

## 2. Materials and Methods

### 2.1. Bacterial Strains and Culture Characteristics

The culture from a blood sample obtained from a male patient in Georgia, United States by the Georgia Department of Public Health was submitted to the SBRL at CDC, Atlanta, Georgia, United States, for identification. Colonies of strain 15‐116A^T^ were recovered following aerobic incubation at 35°C for 5–7 days on trypticase soy (TS) agar supplemented with 5% sheep blood. The closest phylogenetic neighbors, *Streptomyces composti* SBST2‐5^T^, *Streptomyces thermocarboxydovorans* DSM 44296^T^, and *Streptomyces thermospinosisporus* DSM 41779^T^. Strains DSM 44296^T^ and DSM 41779^T^ were obtained from the DSMZ‐German Collection of Microorganisms and Cell Cultures (https://www.dsmz.de/collection/catalogue/microorganisms/catalogue). The strains were maintained in trypticase soy broth (TSB) supplemented with 20% v/v glycerol and stored at −80°C.

Morphological and cultural characters of strain 15‐116A^T^ were recorded following examination of growth on several media: TS agar supplemented with 5% sheep blood, nutrient agar, International Streptomyces Project (ISP) media ISP2, ISP3, ISP4, ISP5, ISP6, and ISP7, GYM (DSMZ Medium 65), and N‐Z amine, as described previously [9, 10]. The ability of the strain to produce diffusible pigments on the media listed above was also recorded. Microscopic cultural features were observed following aerobic incubation at 28°C for 7 days. A 7‐day‐old culture of the strain grown on ISP3 medium at 37°C was examined by light microscopy using a Nikon microscope (Nikon Eclipse Ni) at 100x magnification to determine the ornamentation of the spore chains. The Gram staining properties were carried out as described by Berd [11]. Growth at different temperatures (15°C, 20°C, 25°C, 30°C, 35°C, 40°C, 45°C, and 50°C) and in the presence of sodium chloride (2%, 4%, and 8%) were evaluated as described by Kusuma et al. [12]. The color of aerial and substrate mycelium were determined using color charts [13]. A 7‐day‐old culture prepared in TSB at 28°C was used for all tests listed above. The cell density of the inoculum was equivalent to 5 on the McFarland scale [14]. The tests were carried out in triplicates.

### 2.2. Phylogenetic Analyses Based on 16S rRNA Gene and Genome Sequences

Whole cell genomic DNA extraction was carried out from cells grown in TSB for 5 days at 28°C using the MasterPure DNA purification kit (Epicentre, Wisconsin) following the manufacturer′s instructions. The purified DNA was subjected to PCR‐mediated amplification and sequencing of a 1449‐bp 16S rRNA gene fragment using the procedure previously described by Lasker et al. [15]. BLASTn software (https://www.ncbi.nlm.nih.gov/blast/) was used for comparison of the 16S rRNA gene sequence to GenBank reference sequences. However, the corresponding 16S rRNA gene sequences of *Streptomyces* species validly named were retrieved from the EzBioCloud database [16, 17]. Neighbor‐joining (NJ) [18–21] and maximum‐likelihood (ML) [18, 19, 21, 22] phylogenetic trees were built using MEGA 12 software [21]. ML tree (Figure S1) was constructed using Jukes–Cantor (1969) model [23] and the highest log likelihood (−4.348, 99) was shown. The number of replicates (110) was determined adaptively [21] and was displayed below the branches. The superior log‐likelihood between a NJ tree [18] and a maximum parsimony (MP) tree was used for the selection of the initial tree. A matrix of pairwise distances computed using the Jukes–Cantor (1969) model was applied for NJ tree. The MP tree was performed with a randomly generated starting tree and had the shortest length among 10 MP tree searches. For NJ tree, the sum of branch length = 0.200 was shown. The bootstrap values (1.000 replicates) were shown below the branches [21]. The evolutionary distances were determined using the Kimura 2‐parameter method [24]. The pairwise deletion option was applied to all ambiguous positions for each sequence pair. Thirty nucleotide sequences with 1.455 positions were included in the final dataset.

For the whole genome sequence, genomic DNA was fragmented using a M220 Focused‐ultrasonicator (Covaris, Inc.), and the DNA fragments were then used to construct a genomic library using the NEBNext Ultra DNA kit (New England Biolabs). Paired‐end sequencing (2 × 250 bp) was performed using MiSeq Version 2 500‐cycle reagent kit (Illumina). Illumina reads were cleaned with BBDUK 37.77 and Trimmomatic [25, 26]. Cleaned reads were assembled using Spades 3.13.0 software as described previously [27, 28]. The quality of the genome sequence was assessed via CheckM (v1.2.3) on the Prokaryotic Genome Annotation Pipeline (PGAP) gene set with the *Streptomyces* CheckM marker set [29]. The annotation of the genome was carried out by PGAP in NCBI [30]. A ML tree based on genome sequences was constructed using the Type Strain Genome Server (TYG) server (https://tygs.dsmz.de/) [31]. Average nucleotide identity (ANI), calculated based on MUMmer, was determined using the JSpecies WS Version 5.0.3 (https://jspecies.ribohost.com/jspeciesws/#home) as described by Richter et al. [32]. Digital DNA–DNA hybridization (dDDH) was determined using TYGS as described by Meier‐Kolthoff et al. [33, 34].

### 2.3. Phenotypic and Chemotaxonomic Analyses

Biomass for chemotaxonomic studies of strain 15‐116A^T^ were obtained from 5‐day‐old cultures prepared on TSB at 28°C, and then freeze‐dried after being washed with sterile distilled water [35]. Stereoisomers of diaminopimelic acid [36] and whole cell sugars were conducted using standard chromatographic procedures as described by Lechevalier and Lechevalier [37] and Staneck and Roberts [38]. Isoprenoid quinones were extracted from freeze‐dried biomass and chromatographic analysis and identification were performed by HPLC coupled to a DAD and high‐resolution spectrometer, as described previously [39]. Polar lipids were identified by two‐dimensional TLC as developed by Minnikin et al. [40]. Cellular fatty acids were extracted, saponified, and methylated from wet biomass of 5‐day‐old active cultures, according to standard protocols of the Microbial Identification System (MIDI Inc.; Version 6.1) [41], and the compounds were identified against the ACTIN6 peak naming database. Summed features were resolved, and results were confirmed by GC‐MS as described previously [42].

The ability of the strain to degrade casein, cellulose, chitin, 0.3% elastin, gelatin, guanine, 0.4% hypoxanthine, pectin, and triutin, to reduce nitrate, and to hydrolyze Tween 20, 40, 60, and 80, starch, urea, 0.4% xanthine, and xylan was performed as described by Berd [11], Weyant et al. [43], and Williams et al. [44]. Biochemical and enzymatic profiles of the strain and its closest neighbors, *S. thermocarboxydovorans* DSM 44296^T^ and *S. thermospinosisporus* DSM 41779^T^, were determined using API‐50 CH, API 20 and API‐ZYM strips BioMérieux (France) following the procedure and conditions described by the manufacturer. Minimal inhibition concentrations (MIC) to 11 antimicrobial agents were determined and analyzed using the CLSI [45] interpretative breakpoints using commercially available antimicrobial panels (PML Microbiologicals, Inc.).

In view of the clinical origin of strain 15‐116A^T^, its genome sequence was screened for virulence factor associated gene using Virulence Factor Database (VFDB https://www.mgc.ac.cn/VFs/main.htm) server with BLAST+ v2.17.0 [46], as well as for antimicrobial resistance genes using AMRFinderPlus v4.2.7 (https://bio.tools/amrfinderplus) tools [47]. The genome of the strain was also subjected to genome mining for Biosynthetic Gene Clusters (BGCs) using AntiSMASH v8.0.4 platform (https://antismash.secondarymetabolites.org/#!/start) with relaxed detection strictness setting [48]. The close relatives of the strain, *S. composti* SBST2‐5^T^, *S. thermocarboxydovorans* DSM 44296^T^, and *S. thermospinosisporus* DSM 41779^T^, were included in these analyses.

## 3. Results and Discussion

BLASTn analysis of an almost complete 16S rRNA gene sequence (1449 bp) for strain 15‐116A^T^ showed that it belonged to the genus *Streptomyces* (Figure 1). The closest related species validly named to the strain with 16S rRNA gene sequence similarity above 99.0% were *S. spinoverrucosus* NRRL 14228^T^ (99.1%), *S. lusitanus* NBRC 13464^T^ (99.0%), *S. venetus* CMU‐AB225^T^ (99.0%), and *S. purpurascens* NBRC 13077^T^ (99.0%). Moreover, the 16S rRNA gene sequence of the strain showed sequence similarity values of 89.9% with *Streptomyces levis* NBRC 15423^T^, *Streptomyces harenosi* PRKS01‐65^T^, *Streptomyces fuscus* GXMU‐J15^T^, *Streptomyces speibonae* NRRL B‐24240^T^, *Streptomyces indiaensis* NBRC 13964^T^, *Streptomyces muensis* MBRL 179^T^, and *Streptomyces coerulescens* ISP 5146^T^; values exceed the threshold of 98.65% for prokaryotic species demarcation [50], above which further taxogenomic investigations (e.g., dDDH and ANI) are required to species‐level assignment of the bacterial strain. The 16S rRNA gene data were not coherent with the phylogenetic position of strain 15‐116A^T^ in ML and NJ phylogenetic trees. The strain formed a distinct branch loosely associated with a subcluster encompassing the type strains listed above (>98.65%) (Figure S1). The poorly supported phylogenetic relationship of the strain within the evolutionary radiation of the genus *Streptomyces* highlighted the low resolution of the 16S rRNA gene to accurately distinguish between closely related species. It has been shown that the 16S rRNA gene sequence has good resolution for genus level identification, but is insufficient for reliable species assignments [51]. These results were expected as the genus have more than 800 species validly named with several closely related lineages that showed highly conserved 16S rRNA gene sequence (99%–100%) (https://lpsn.dsmz.de/), though their genomic and phenotypic difference [52, 53]. Therefore, genome‐based phylogeny and comparative genomic analyses were carried out. The genome of strain 15‐116A^T^ has a high quality with 97.9% completeness (> 90%) and 0.6% contamination (< 5%), meeting the required standards for applying genome in prokaryotic taxonomy [54]. Strain 15‐116A^T^ had a genome size of 7.3 Mb with a G + C content of 71%, gene quantity of 7082, and protein coding sequences of 6615. These genomic features were coherent with those of the genus *Streptomyces* (5.96–12.01 Mb and G + C content of 71%–74% [mol%]) [55], and distinguished the strain from its close relatives, as shown in Table 1.

**Figure 1:**
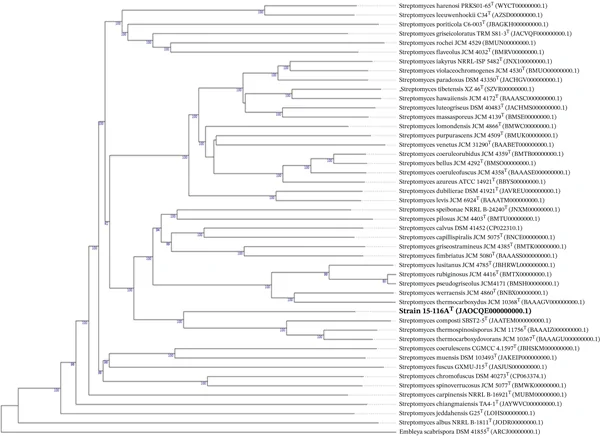
Genome Blast Distance Phylogenetic (GBDP) tree inferred with FastME 2.1.6.1 [49] showing the phylogenetic relationship of strain 15‐116A^T^ within the evolutionary radiation of the genus *Streptomyces*. The branch lengths are scaled in terms of GBDP distance formula *d*_5_. The numbers above branches are GBDP pseudo‐bootstrap support values > 60% from 100 replications, with an average branch support of 92.4%. *Embleya scabrispora* DSM 41855^T^ was used as an outgroup.

**Table 1:** Genomic features of strain 15‐116A^T^ and its close phylogenomic relatives.

	Strain 15‐116A^T^	*Streptomyces composti* SBST2‐5^T^	*S. thermocarboxydovorans* JCM 10367^T^	*S. thermospinosisporus* JCM 11756^T^
Genome size (Mb)	7.3	6.5	6.5	6.9
GC content (%)	71.0	72.0	71.5	72.0
Number of contigs	560	100	190	300
Contig N50 (kb)	21.7	166.8	79.4	42.4
Contig L50	94	16	29	55
Genes	7082	5820	6191	6471
Protein‐coding	6615	5516	6075	6353
Completeness (%)	97.9	99.81	99.87	99.56
Contamination (%)	0.6	0.09	2.29	0.38
Genome accession number	JAOCQE000000000.1	JAATEM000000000.1	BAAAGU000000000.1	BAAAIZ000000000.1

Genome‐based phylogeny is inferred from the analysis of thousands of genes, reflecting overall genomic evolution and providing higher evolutionary resolution and reliable species‐level relationships. In the Genome Blast Distance Phylogenetic tree (GBDP), strain 15‐116A^T^ occupied a well‐supported distinct branch closely associated with a subcluster housing the type strains of *S. composti*, *S. thermocarboxydovorans*, and *S. thermospinosisporus* and distant from its close neighbors retrieved based on the 16S rRNA gene (Figure 1). In the phylogenomic tree, *S. composti* appeared to be the closest relative to strain 15‐116A^T^, whereas *S. thermocarboxydovorans* and *S. thermospinosisporus* showed a very close evolutionary relationship. These results were in line with the comparative genomic analyses.

The dDDH and ANI values between strain 15‐116A^T^ and its closest relatives, *S. composti* SBST2‐5^T^, *S. thermospinosisporus* JCM 11756^T^, and *S. thermocarboxydovorans* JCM 10367^T^, were 35.9%, 37.3%, and 37.1%, and 89.6%, 90.1%, and 90.2%, respectively (Table 2); values well below the thresholds of 70% (DDH) and 95%–96.7% (ANI) for prokaryotic species demarcation [56–59]. In view of the close phylogenetic relationship of *S. thermocarboxydovorans* JCM 10367^T^ and *S. thermospinosisporus* JCM 11756^T^ in GBDP tree, the taxonomic status of these species was assessed. The dDDH and ANI values between the genome sequence of strains JCM 11756^T^ and JCM 10367^T^ were 72.3% and 96.7%, respectively; values above the defined thresholds for prokaryotic species demarcation and within the range of 70%–79% (dDDH) for subspecies demarcation. The phylogenetic convergence of these species was coherent with the 16S rRNA gene sequence identity value of 99.9% and their close phylogenomic relatedness (Figures 1 and S1). Therefore, *S. thermospinosisporus* is considered a later heterotypic synonym of *S. thermocarboxydovorans* and is classified as a subspecies of *S. thermocarboxydovorans*, for which the name *S. thermocarboxydovorans* subsp. *thermospinosisporus* subsp. nov. is proposed. This conclusion is in concordance with the observed phenotypic similarities between the two taxa and is also supported by their distinct characteristics. Both *S. thermocarboxydovorans* and *S. thermocarboxydovorans* exhibit rectiflexible spore chains, though they can be differentiated by spore surface morphology; *S. thermocarboxydovorans* forming smooth spore surfaces, whereas *S. thermospinosisporus* displaying spiny spore surfaces. Moreover, the type strain of *S. thermospinosisporus* was able to use mannitol, raffinose, and sucrose as sole carbon sources and grew in the presence of phenol, unlike *S. thermocarboxydovorans* [60, 61].

**Table 2:** Digital dDDH and ANI values between the genome sequence of strain 15‐116A^T^ and those of its closest phylogenetic relatives in the genus *Streptomyces*.

Subject strain	dDDH (%)	ANI (%)	Genome accession number
*Streptomyces azureus* ATCC 14921^T^	29.1	87.3	BBYS00000000.1
*Streptomyces calvus* DSM 41452	31.8	88.4	CP022310.1
*Streptomyces capillispiralis* JCM 5075^T^	32.1	88.5	BNCE00000000.1
*Streptomyces carpinensis* NRRL B‐16921^T^	27.5	86.9	MUBM01000001.1
*Streptomyces chiangmaiensis* TA4‐1^T^	25.3	86.0	JAYWVC010000001.1
*Streptomyces chromofuscus* DSM 40273^T^	27.9	87.0	CP063374.1
*Streptomyces coeruleofuscus* JCM 4358^T^	29.0	87.3	BAAASE000000000.1
*Streptomyces bellus* JCM 4292^T^	29.2	87.3	CP137524.1
*Streptomyces coerulescens* CGMCC 4.1597^T^	27.2	86.6	JBHSKM010000001.1
*Streptomyces composti* SBST2‐5^T^	35.9	89.6	JAATEM000000000.1
*Streptomyces dubilierae* DSM 41921^T^	29.0	87.4	JAVREU010000001.1
*Streptomyces fimbriatus* JCM 5080^T^	33.4	89.1	BAAASS000000000.1
*Streptomyces flaveolus* JCM 4032^T^	28.2	87.2	BMRV01000001.1
*Streptomyces fuscus* GXMU‐J15^T^	27.1	86.8	JASJUS010000001.1
*Streptomyces griseicoloratus* TRM S81‐3^T^	28.8	87.4	JACVQF010000001.1
*Streptomyces griseostramineus* JCM 4385^T^	33.0	88.9	BMTK01000001.1
*Streptomyces griseomycini* JCM 4382^T^	33.2	88.9	BMTI00000000.1
*Streptomyces harenosi* PRKS01‐65^T^	28.7	87.3	WYCT01000001.1
*Streptomyces hawaiiensis* JCM 4172^T^	28.4	87.1	BAAASC010000001.1
*Streptomyces iakyrus* NRRL ISP‐5482^T^	28.6	87.2	JNXI01000001.1
*Streptomyces jeddahensis* G25^T^	25.1	86.0	LOHS01000001.1
*Streptomyces leeuwenhoekii* C34^T^	28.5	87.3	AZSD01000001.1
*Streptomyces levis* JCM 6924^T^	29.0	87.4	BAAATM000000000.1
*Streptomyces lomondensis* JCM 4866^T^	29.4	87.5	BMWC00000000.1
*Streptomyces lusitanus* JCM 4785^T^	29.4	87.7	JBHRWL000000000.1
*Streptomyces massasporeus* JCM 4139^T^	28.4	87.1	BMSE01000001.1
*Streptomyces muensis* DSM 103493^T^	27.6	86.8	JAKEIP010000001.1
*Streptomyces paradoxus* DSM 43350^T^	28.6	87.2	JACHGV010000001.1
*Streptomyces pilosus* JCM 4403^T^	30.6	87.9	BMTU00000000.1
*Streptomyces poriticola* C6‐003^T^	27.8	87.0	JBAGKH010000001.1
*Streptomyces pseudogriseolus* JCM 4171^T^	29.3	87.7	BMSH01000001.1
*Streptomyces purpurascens* JCM 4509^T^	28.7	87.2	BMUK01000001.1
*Streptomyces speibonae* NRRL B‐24240^T^	31.8	88.3	JNXM00000000.1
*Streptomyces spinoverrucosus* JCM 5077^T^	27.6	86.9	BMWK01000001.1
*“Streptomyces tibetensis”* XZ 46	28.4	87.1	SZVR01000001.1
*Streptomyces thermocarboxydovorans* JCM 10367^T^	37.1	90.2	BAAAGU000000000.1
*Streptomyces thermocarboxydus* JCM 10368^T^	29.3	87.7	BAAAGV000000000.1
*Streptomyces thermospinosisporus* JCM 11756^T^	37.3	90.1	BAAAIZ000000000.1
*Streptomyces venetus* JCM 31290^T^	28.7	87.2	BAABET010000001.1
*Streptomyces rochei* JCM 4529	28.1	87.3	BMUN01000001.1
*Streptomyces violaceochromogenes* JCM 4530^T^	28.4	87.2	BMUO01000001.1
*Streptomyces werraensis* JCM 4860^c^	29.4	87.6	BNBX00000000.1

The authenticity of the type strain of these species was checked by comparing the original 16S rRNA gene sequence generated by PCR reaction with their respective sequences derived from the genome sequences. The results revealed that the aligned 16S rRNA gene sequences (PCR) of *S. thermocarboxydovorans* DSM 44296^T^ (Accession Number: U94489) and *S. thermocarboxydovorans* NBRC 16324^T^ (Accession Number: AB249929) showed a similarity value of 99.4% (~9 nucleotides difference), which questioned the quality of the 16S rRNA gene sequence and/or the authenticity of the type strain *S. thermocarboxydovorans.* However, the 16S rRNA gene sequences of *S. thermocarboxydovorans* DSM 44296^T^ and that of the genome of *S. thermocarboxydovorans* JCM 10367^T^ have a similarity value of 99.9%. Strains DSM 44296^T^ and JCM 10367^T^ were obtained from the authors who described this species, unlike strain NBRC 16324^T^. These results questioned the quality of the 16S rRNA gene sequence and/or the authenticity of strain NBRC 16324^T^. The 16S rRNA gene sequences of *S. thermospinosisporus* AT10^T^ (Accession Number: AF333113) and NBRC 100043^T^ (Accession Number: AB249914.1) were identical. In view of these data, *S. thermospinosisporus* should be considered as a subspecies of *S. thermocarboxydovorans*, for which the name *S. thermocarboxydovorans* subsp. *thermospinosisporus* subsp. nov., is proposed. Consequently, a nominotypical subspecies, *S. thermocarboxydovorans* subsp. *thermocarboxydovorans*, is created, and an emended description of the *S. thermocarboxydovorans* is provided.

Strain 15‐116A^T^ is a Gram‐stain positive, acid‐fast stain negative, aerobic, and nonmotile actinobacterial strain. It produced rectiflexible spore chains (Figure S2). Colonies cultured on TS agar with 5% sheep blood were elevated, tan, brown on bottom of plate, and showed no hemolysis. After 5 days of incubation at 28°C, strain 15‐116A^T^ formed orange substrate mycelium on ISP1, ISP4, ISP5, and ISP6, whereas it acquired orange brownish substrate mycelium on GYM, ISP2, ISP7, and N‐Z‐amine agar media. However, it developed beige grayish to gray aerial mycelium on ISP3 medium. Growth on GYM, ISP2 agar produced brown colonies and a diffusible pigment. Good growth of the strain was observed on ISP2, ISP3, ISP6, ISP7, GYM, and N‐Z‐amine agar. It was able to grow in the presence of up to 4% NaCl, between 20°C–40°C, but not at temperature ≥ 45°C on ISP2 agar.

The combination of morphological and phenotypic traits allowed strain 15‐116A^T^ to be distinguished from the type strains of its closest phylogenetic neighbors. Strain 15‐116A^T^ was able to metabolize D‐adonitol, L‐rhamnose, D‐melibiose, and D‐saccharose, but unable to assimilate D‐ribose and phenylacetic acid as sole carbon sources, unlike its close relatives strains DSM 41779^T^ and DSM 44296^T^. Strain 15‐116A^T^ shared several biochemical and enzymatic characteristics with *S. composti* but could be distinguished by its ability to produce *α*‐mannosidase, whereas *S. composti* was able to assimilate D‐xylitol and D‐raffinose (Table 3).

**Table 3:** Phenotypic features differentiating strain 15‐116^T^ from its closest phylogenetic neighbors.

	Strain 15‐116A^T^	*S. composti* SBST2‐5^Ta^	*S. thermocarboxydovorans* DSM 44296^T^	*S. thermospinosisporus* DSM 41779^T^
Adipic acid	(+)	na	+	+
D‐Adonitol	+	na	−	−
L‐Arabinose	+	+	(+)	−
L‐Arginine	−	na	+	−
D‐Cellobiose	+	+	+	+
Dulcitol	−	na	−	−
D‐Galactose	+	+	+	+
D‐Mannitol	+	+	+	+
D‐Mannose	+	+	+	(+)
D‐Melibiose	+	+	−	−
D‐Melezitose	+	na	−	+
Methyl‐a‐D‐glucopyranoside	+	na	+	−
D‐Raffinose	−	+	−	−
L‐Rhamnose	+	na	−	−
D‐Ribose	−	na	+	+
D‐Saccharose (sucrose)	+	+	−	+
D‐Trehalose	+	+	+	+
D‐Turanose	+	na	−	+
Phenylacetic acid	−	na	+	+
Potassium 5‐ketogluconate	−	na	(+)	−
Potassium 2‐ketogluconate	−	na	−	(+)
Trisodium citrate	(+)	na	−	−
Urea	(+)	na	+	−
D‐Xylitol	−	+	−	−
D‐Xylose	+	+	+	+
Hypoxanthine decomposition	+	+	nt	nt
Enzymatic features				
Alkaline phosphatase	+	na	(+)	+
*α*‐Chymotrypsin, trypsin	(+)	−	(+)	+
Esterase, esterase lipase, ß‐glucuronidase	(+)	na	+	+
Esterase lipase (C8)	−	−	−	−
*α*‐Galactosidase	(+)	na	(+)	−
ß‐Galactosidase	+	+	+	+
ß‐Glucosidase	+	na	+	−
N‐Acetyl‐ß‐glucosaminidase	+	na	(+)	+
Lipase (C 14)	−		+	−
*α*‐Mannosidase	+	−	(+)	+
Naphthol‐AS‐BI‐phosphohydrolase	(+)	−	+	+
Chemotaxonomic features				
DAP	*LL*‐DAP	*LL*‐DAP	*LL*‐DAP	*LL*‐DAP
Whole cell sugars	Glu, Rib, and traces of Gal and Mann	Gal, Glu, Mann, and Rib	na	na
Predominant menaquinones (> 10%)	MK‐9(H_6_) and MK‐9(H_8_)	MK‐9(H_6_) and MK‐9(H_8_)	na	MK‐9(H_8_)^c^
Polar lipid profile	DPG, PE, PI, PL, L	DPG, PE, PG, PI, PIM, PLs_1-3_, AL	na	DPG, PE^c^
Major fatty acids (> 10%)	*iso-*C_15:0_, *anteiso-*C_15:0_, *iso-*C_16:0_, *anteiso-*C_17:0_	*iso*‐C_15:0_, *iso*‐C_16:0_, *anteiso*‐C_15:0_	*iso-*C_15:0_, *anteiso-*C_15:0_, *iso-*C_16:0_^b^	na

*Note:* “+,” positive; “−,” negative; “(+),” weak reaction. All strains (except *S. Composti* SBST2‐5^T^) were able to metabolize N‐acetylglucosamine, amygdalin, D‐arabitol, arbutin, esculin, D‐fructose, gentiobiose, D‐glucose, glycerol, glycogen, inositol, D‐lactose (bovine origin), malic acid, D‐maltose, potassium gluconate, salicin, and starch (Amidon). Able to reduce potassium nitrate, hydrolyse gelatin, produced acid phosphatase, cystine arylamidase, *α*‐glucosidase, 4‐nitrophenyl‐ß‐D‐galactopyranoside, leucine arylamidase, and valine arylamidase. All strains were unable to assimilate D‐arabinose, L‐arabitol, caprices acid, erythritol, D‐fucose, L‐fucose, inulin, D‐lyxose, methyl‐a‐D‐mannopyranoside, methyl‐ß‐D‐xylopyranoside, L‐sorbose, D‐sorbitol, D‐tagatose, and L‐xylose. Unable to ferment glucose and produce indole (tryptophan) and *α*‐fucosidase.

Abbreviations: AL, aminolipid; DPG, diphosphatidylglycerol; gal, galactose; Glu, glucose; L, lipid; Mann, mannose; na, data not available; nt, not tested; PE, phosphatidylethanolamine; PG, phosphatidylglycerol; PI, phosphatidylinositol; PL, phospholipid; PIM, phosphatidylinositol mannoside; rib, ribose.

^a^Data taken from Duangupama et al. [62].

^b^Data taken from Law et al. [63].

^c^Data taken from Kämpfer [9].

The chemotaxonomic characters of strain 15‐116A^T^ were consistent with the genus *Streptomyces* [5, 6]. The strain was characterized by the presence of *LL*‐DAP in its peptidoglycan; glucose, ribose, and traces of galactose and mannose as whole cell sugars. A similar sugar profile was described for its closest neighbor *S. composti* SBST2‐5^T^ [62]. The polar lipid profile of the strain consisted of diphosphatidylglycerol (DPG), phosphatidylethanolamine (PE), and phosphatidylinositol (PI). Phospholipid (PL) and lipid (Figure S3) while its closest neighbor *S. composti* SBST2‐5^T^ had in addition phosphatidylglycerol (PG) [62]. Predominant menaquinones were MK‐9(H_8_) (> 65%) and MK‐9(H_6_) (> 15%), like strain SBST2‐5^T^ (Table 3) [62]. The major fatty acids (> 10%) of strain 15‐116A^T^ were *iso-*C_15:0_, *anteiso*‐C_15:0_, *iso*‐C_16:0_, and C_17:0_ *anteiso* (Table S1, Table 3); coherent with strain SBST2‐5^T^ [62] (Table 3).

Antimicrobial susceptibility tests showed strain 15‐116A^T^ was resistant to ceftriaxone but was susceptible to amikacin, ampicillin, amoxicillin/clavulanate, clarithromycin, imipenem, linezolid, minocycline, moxifloxacin, trimethoprim/sulfamethoxazole, and vancomycin.

No antimicrobial resistance genes were detected in the genome of the strain and neither its closest relatives, *S. composti* SBST2‐5^T^, *S. thermocarboxydovorans* DSM 44296^T^, and *S. thermospinosisporus* DSM 41779^T^, which can be explained by the fact that AMRFinderPlus platform is designed more for Gram‐negative enteric bacteria than Gram‐positive actinomycetes. Genome screening for virulence factor associated genes showed that the strain harbored a gene encoding for isocitrate lyase, which is required for persistent infection of *Mycobacterium tuberculosis* (YP_177728) [64]. The strain had gene clusters associated with phenazine biosynthesis (NP_252902), such as pyocyanin, that can cause host tissue damage and interfere with immune cell function leading to worsening the disease [65]. These genes were absent in the genome sequences of the close relatives, strains DSM 44296^T^, DSM 44293^T^, and SBST2‐5^T^. However, the latter and strain 15‐116A^T^ possessed in their genome a gene encoding Tu elongation factor (WP_003028672), which plays important role in the bacterium′s ability to adhere to host cells [66]. As most of *Streptomyces* strains, the strain harbored numerous BGCs encoding various classes of secondary metabolites, many of which were only present in the genome of strain15‐116A^T^, as shown in Table S2.

## 4. Conclusion

Phenotypic, genotypic, and genomic features of this rare clinical strain 15‐116A^T^ were consistent with the genus *Streptomyces* and could be clearly distinguish it from other closely related species. Therefore, we propose strain 15‐116A^T^ as the type strain of a novel species within the genus *Streptomyces* and thereby propose the name *Streptomyces sanguinis* sp. nov.

### 4.1. Taxonomic Consequences

#### 4.1.1. Description of *S. sanguinis* sp. nov.

*sanguinis* (san′gui.nis L. masc. n. *sanguinis*, blood; L. gen. masc. n. *sanguinis*, of the blood).

An aerobic, Gram‐stain positive, nonmotile actinobacterium that produces branched mycelium and aerial hyphae bearing spore chains of rectiflexibilis type. Forms orange substrate mycelium on ISP1, ISP4, ISP5, and ISP6 while it acquired orange brownish substrate mycelium on GYM, ISP2, ISP7, and N‐Z‐amine agar media. Develops a beige grayish to gray aerial mycelium on ISP3 medium. It forms brown colonies and produces a diffusible pigment on GYM and ISP2 agar. It is able to grow in the presence of up to 4% NaCl. Colonies grown on TS agar supplemented with 5% sheep blood are elevated tan, brown on bottom of plate, with no hemolysis. Optimal growth of the strain is observed on TS agar with 5% sheep blood, GYM, and ISP2 at 28°C and 37°C, and at pH 7. Positive for urea hydrolysis and nitrate reduction. Degrades gelatin, Tween 20, tribtin, elastine (0.3%), guanine, and hypoxanthine (0.4%), but not Tween 40, Tween 60, casein, cellulose, chitin, or pectine. Utilizes N‐acetylglucosamine, D‐adonitol, amygdalin, L‐arabinose, D‐arabitol, arbutin, D‐cellobiose, esculin, D‐fructose, D‐galactose, gentiobiose, methyl‐a‐D‐glucopyranoside, D‐glucose, glycerol, glycogen, inositol, D‐lactose (bovine origin), malic acid, D‐maltose, D‐mannitol, D‐mannose, D‐melibiose, D‐melezitose, potassium gluconate, L‐rhamnose, D‐saccharose (sucrose), salicin, starch (amidon), D‐trehalose, D‐turanose, and D‐xylose. Able to reduce potassium nitrate, hydrolyse gelatin, produced ß‐Galactosidase, 4‐nitrophenyl‐ß‐D‐galactopyranoside, ß‐glucosidase, N‐acetyl‐ß‐glucosaminidase, acid phosphatase, cystine arylamidase, *α*‐glucosidase, *α*‐mannosidase, leucine arylamidase, and valine arylamidase. Grows in presence of nalidixic acid, lithium chloride, and potassium tellurite. Polar lipids pattern consisted of DPG, PE, PI, PL, and an unknown lipid (L). Whole cell hydrolysates include *LL*‐A_2_pm and glucose, ribose, and traces of galactose and mannose. Predominant menaquinones (> 25%) are MK‐9(H_8_) and MK‐9(H_6_). The major fatty acids (>1 0%) consist of *iso-*C_15:0_, *anteiso-*C_15:0_, *iso-*C_16:0_, and *anteiso-*C_17:0_. The type strain has a genome size of 7.3 Mbp and a DNA G + C content of 71.0%.

The type strain 15‐116A^T^ (= DSM 106436^T^ = ATCC BAA‐2935^T^) was isolated from the blood of a 31‐year‐old male patient hospitalized in the state of Georgia, United States. The GenBank accession numbers of the 16S rRNA gene and draft genome sequences are MH553378 and JAOCQE010000000, respectively.

#### 4.1.2. Description of *S. thermocarboxydovorans* subsp. *thermocarboxydovorans* subsp. nov.

*thermocarboxydovorans* (http://ther.mo.car.bo.xy.do'vo.rans. Gr. fem. adj. *thermê*, heat; N.L. neut. adj. *carboxydum*, carbon monoxide; L. v. *voro*, to eat greedily, devour; N.L. masc. part. adj. *thermocarboxydovorans*, thermophilic, carbon monoxide consuming).

The description is as given by Kim et al. [61]. Type strain is AT52, CIP 105544, DSM 44296, IFO 16324, JCM 10367, KCTC 993, NBRC 16324, NCIMB 13530, and NRRL B‐24317. The genome size of the type strain is 6.5 Mb and a DNA G + C content of 71.5%. The GenBank accession number of the genome is BAAAGU000000000.1.

#### 4.1.3. Description of *S. thermocarboxydovorans* subsp. *thermospinosisporus* subsp. nov.

*thermospinosisporus* (http://ther.mo.spi.no.si'spo.rus. Gr. masc. adj. *thermos*, hot; L. masc. adj. *spinosus*, thorny; N.L. masc. adj. suff. *-sporus*, a seed; N.L. masc. adj. *thermospinosisporus*).

#### 4.1.4. Basonym: *S. thermospinosisporus* (corrig. Kim and Goodfellow [60])

The description is as given by Kim and Goodfellow [60]. The type strain is AT10, BCRC 16367, DSM 41779, JCM 11756, KCTC 9909, NBRC 100043, and NRRL B‐24318. The genome size of the type strain is 6.9 Mb and a DNA G + C content of 72.0%. The GenBank accession number of the genome is BAAAIZ000000000.1.

#### 4.1.5. Emended Description of *S. thermocarboxydovorans* Kim et al. [61]

The description is as given by Kim et al. [61], with the following modification after inclusion of *S. thermospinosisporus* [49] as a subspecies: facultatively rectiflexible spore chains with smooth or spiny surfaces; able to use mannitol, raffinose, and sucrose as sole carbon sources; and resistant to phenol.

## Author Contributions

I.N. and B.A.L: conceptualization, investigation, and writing original draft. J.W., M.N‐S., C.A.G., and J.R.M: investigation, methodology, formal analysis, and data curation.

## Funding

Open Access funding was enabled and organized by Projekt DEAL.

## Disclosure

All authors read and approved the final version of the manuscript. The findings and conclusions in this report are those of the authors and do not necessarily represent the official position of the Centers for Disease Control and Prevention. The 16S rRNA gene and genome sequences of strain DSM 15‐116A^T^ have been deposited in the GenBank under Accession Numbers MH553378 and JAOCQE010000000, respectively.

## Ethics Statement

This novel species of *Streptomyces* was identified and characterized as part of routine reference laboratory practice and has been confirmed by CDC as nonresearch. As such, the activities described herein did not require the review and approval of the CDC IRB. [See e.g., 45 C.F.R. part 46, 21 C.F.R. part 56; 42 U.S.C. §241(d); 5 U.S.C. §552a; 44 U.S.C. §3501 et seq.]

## Conflicts of Interest

The authors declare no conflicts of interest.

## Supporting information


**Supporting Information** Additional supporting information can be found online in the Supporting Information section. Figure S1: (a) Neighbor‐joining and (b) maximum likelihood phylogenetic trees, based on the 16S rRNA gene sequences showing the phylogenetic relationship of strain 15‐116A^T^ with its closely related species. For (a) NJ tree, the bootstrap percentages based on 1000 resampled datasets are shown below the branches (only values ≥ 60%). For (b) ML, the number of replicates (110) was determined adaptively and displayed below the branches. The tree was rooted with *Streptacidiphilus albus* JL83^T^ as an outgroup. Figure S2: Light micrograph of rectiflexibile spore chains of strain 15‐116A^T^ grown on ISP3 medium for 7 days at 37°C. Figure S3: Two‐dimensional TLC plate of polar lipids extracted from isolate 15‐116A^T^ stained with (a) molybdatophosphoric acid (SigmaP1518); (b) molybdenum blue; (c) ninhydrin; (d) *α*‐Naphthol reagent; and (e) Dragendorff′s reagent. Key: DPG, diphosphatidylglycerol; PE, phosphatidylethanolamine; PI, phosphatidylinositol; PL, phospholipid; L, lipid. Solvent 1: chloroform: methanol: distilled water (65:25:4 v/v/v); Solvent 2: chloroform: glacial acetic acid: methanol: distilled water (80:12:15:4 v/v/v). Table S1: Fatty acid profiles of strain 15‐116A^T^. Only fatty acids ≥ 4 were listed in the table.  ^∗^Data taken from Duangupama et al. [62]. Table S2: Biosynthetic gene cluster predictions using antiSMASH v8.0.4. Features that differentiate strain 15‐116AT are first, and features that are shared among near neighbors are at the bottom of the table.

## Data Availability

The data that support the findings of this study are openly available in National Center for Biotechnology Information at https://www.ncbi.nlm.nih.gov/.
