# Supplementary material for: Streptomyces sanguinis sp. nov., a Ceftriaxone‐Resistant Strain Obtained From Human Blood and Reclassification of Streptomyces thermospinosisporus as Streptomyces thermocarboxydovorans subsp. thermospinosisporus subsp. nov
Source: Int J Microbiol. 2026 Oct 2;2026:5214822. doi: 10.1155/ijm/5214822 (PMC13633455; doi:10.1155/ijm/5214822)
Supplement: Supplementary file 1 [file IJM-2026-5214822-s001.docx]

Imen Nouioui^1*^, Jacqueline Wolf^1^, Meina Neumann-Schaal^1^, Christopher A. Gulvik^2^, John R. McQuiston^2^, Brent A. Lasker^2^

^1^Leibniz Institute DSMZ, German Collection of Microorganisms and Cell Cultures, Braunschweig, 38124, Germany

^2^Bacterial Special Pathogens Branch, Division of High-Consequence Pathogens and Pathology, National Center for Emerging and Zoonotic Infectious Disease, Centers for Disease Control and Prevention, Atlanta, GA 30329

**ORCIDs**:

JW: 0000-0003-0672-6999

MNS: [0000-0002-1641-019X](https://orcid.org/0000-0002-1641-019X)

BAL: 0000-0002-6480-0627

CAG: [0000-0001-8754-9829](https://orcid.org/0000-0001-8754-9829)

JRM: [0000-0002-6502-6872](https://orcid.org/0000-0002-6502-6872)

IN: [0000-0002-7973-7640](https://orcid.org/0000-0002-7973-7640)

**Section:** Actinobacteria

**Key words** *Actinomycetota,* BGCs, systematics, virulence factors*, Pathogens*

*Correspondence: Imen Nouioui, imen.nouioui@dsmz.de

b

a

**Figure S1.** Neighbour-joining (a) and maximum likelihood (b) phylogenetic trees, based on the 16S rRNA gene sequences showing the phylogenetic relationship of strain 15-116A^T^ with its closely related species. For NJ tree (a), the bootstrap percentages based on 1,000 resampled data sets are shown below the branches (only values ≥60%). For ML (b), the number of replicates (110) was determined adaptively and displayed below the branches. The tree was rooted with *Streptacidiphilus albus* JL83^T^ as an outgroup.

**Figure S2.** Light micrograph of rectiflexibile spore chains of strain 15-116A^T^ grown on ISP3 medium for 7 days at 37°C.

**Figure S3.** Two-dimensional TLC plate of polar lipids extracted from isolate 15-116A^T^ stained with (a) molybdatophosphoric acid (SigmaP1518); (b) molybdenum blue ; (c) ninhydrin**;** (d) α-Naphthol reagent; and (e) Dragendorff's reagent. Key: DPG, diphosphatidylglycerol; PE, phosphatidylethanolamine; PI, phosphatidylinositol; PL, phospholipid; L, lipid. Solvent1: chloroform: methanol: distilled water (65:25:4 v/v/v/); solvent 2: chloroform: glacial acetic acid: methanol: distilled water (80:12:15:4 v/v/v).

**Table S1.** Fatty acid profiles of strain 15-116A^T^

| **Fatty acid profiles** | **Strain 15-116A^T^** | ***S. composti*** **SBST2-5^T^***** |
| --- | --- | --- |
| *iso-*C_14:0_ | - | 10.0 |
| C_15:0_ | - |  |
| C_15:0_ *iso* | 10.4 | 27.4 |
| C_15:0_ *anteiso* | 17.5 | 15.7 |
| C_16:0_ *ıso* H | - |  |
| C_16:0_ *ıso* | 20.1 | 23.0 |
| C_16:1_ cıs 9 | 4.9 |  |
| C_16:0_ | 6.0 | 5.1 |
| C_16:0_ 9? methyl | 5.7 | - |
| C_17:1_ *anteiso* C | 6.9 | - |
| C_17:0_ *anteiso* | 12.8 | - |
| *iso*-C_17:0_ | - | 4.4 |

Only fatty acids ≥4 were listed in the table. ******* data taken from Duangupama et al. [60].

**Table S2.** Biosynthetic gene cluster predictions using antiSMASH v8.0.4. Features that differentiate strain 15-116A^T^ are first, and features that are shared among near neighbours are at the bottom of the table.

| **Biosynthetic Gene Cluster Type** | **Most Similar Known Cluster Product** | **Strain 15-116A^T^** | ***S. composti*** | ***S. thermocarboxydovorans*** | ***S. thermospinosisporus*** |
| --- | --- | --- | --- | --- | --- |
|  |  |  | **SBST2-5^T^** | **JCM 10367^T^** | **JCM 11756^T^** |
| Aminopolycarboxylic Acid Metallophores | Ethylenediaminesuccinic acid hydroxyarginine | high | - | - | - |
| Butyrolactone,NRPS-like,Beta-Lactone,NRPS | Griseoviridin/Fijimycin A | low | - | - | - |
| Lanthipeptide Class I |  | high | - | - | - |
| NRPS, Type I | JBIR-126 | high | - | - | - |
| NRPS, T3PKS, & transAT-PKS | Guangnanmycin | medium | - | - | - |
| T2PKS | Resistomycin/Resistoflavine | high | - | - | - |
| RiPP-like | Streptamidine | medium | - | - | - |
| NRPS,T1PKS,terpene | Tirandamycin | - | high | - | - |
| Terpene | Isorenieratene | - | - | high | - |
| NRPS | Cyclofaulknamycin | - | - | - | medium |
| Hydrogen Cyanide | Aborycin | low | low | - | low |
| Indole | Azodyrecin A/Azodyrecin B/Azodyrecin C | low | low | - | - |
| Lanthipeptide class III (Class III lanthipeptides) | SapB | high | - | - | - |
| Terpene Precursor |  | - | high | - | - |
| Ectoine | Ectoine | high | high | high | high |
| Terpene | Albaflavenone | high | high | high | high |
| Terpene | Geosmin | high | high | high | high |
| Terpene | Hopene | high | medium | medium | medium |
| Terpene | Isorenieratene | high | high | high | high |
| NRPS-independent Siderophores | Desferrioxamin B/Desferrioxamine E | high | high | high | high |
| NRPS-independent Siderophores | Kinamycin | low | low | low | low |
| RiPP-like | Informatipeptin | low | low | low | low |
| Type II Polyketide Synthase (T2PKS) | Spore pigment | high | high | high | high |

- Not detected
